# Spinal cord stimulation using time-dynamic pulses achieves longer reversal of allodynia compared to tonic pulses in a rat model of neuropathic pain

**DOI:** 10.3389/fpain.2025.1541078

**Published:** 2025-04-09

**Authors:** Changfang Zhu, Ki-Soo Jeong, Muhammad Edhi, Victoria Rogness, Carl Y. Saab, Rosana Esteller

**Affiliations:** ^1^ Research and Development, Boston Scientific Neuromodulation, Valencia, CA, United States; ^2^ Biomedical Engineering, Cleveland Clinic Lerner Research Institute, Cleveland, OH, United States; ^3^ School of Engineering, Brown University, Providence, RI, United States; ^4^ Internal Medicine, University of Buffalo, Buffalo, NY, United States; ^5^ School of Medicine, Case Western Reserve University, Cleveland, OH, United States

**Keywords:** neuromodulation, dynamic, pattern, wash-in, wash-out, sigmoidal, double-sigmoid, model fit

## Abstract

**Background:**

Spinal cord stimulation (SCS) utilizing time-dynamic pulses (TDPs) is an emergent field of neuromodulation that continuously and automatically modulates pulse parameters. We previously demonstrated that TDPs delivered for 60 min at paresthesia-free or minimal paresthesia amplitudes significantly reversed allodynia in a rat model of neuropathic pain. Because the anti-allodynic effect was observed to persist post-stimulation, we hypothesized that the anti-nociceptive effects of TDPs may persist longer than those of tonic stimulation.

**Methods:**

We extended SCS stimulation period up to 90 min and investigated the temporal dynamics of SCS-induced analgesia through PWT analysis of the aggregated data from both cohorts.

**Results:**

Both TDPs and tonic stimulation reversed paw withdrawal thresholds (PWT) to near pre-neuropathic levels within 30 min. Most TDPs exhibited significantly slower ramp-up slope (analgesia ‘wash-in' rates) as compared to tonic stimulation. All TDPs showed slower wind-down slopes (analgesia ‘wash-out’ rates) compared to tonic, with pulse width modulation reaching significance. Extending SCS from 60 to 90 min revealed that all TDPs maintained analgesic efficacy longer than tonic stimulation, which showed significant decrease at both 75 and 90 min.

**Discussion:**

Although TDPs and tonic stimulation comparably mitigated allodynia, TDPs exhibited slower rate of wash-out, suggesting longer-lasting analgesic effects and potentially different mechanisms of action.

## 1 Introduction

Neuromodulation is the manipulation of neural activity through targeted delivery of electromagnetic, ultrasound, optical or other stimuli or pharmacological agents that modulate the activity of the nervous system, thus offering a wide range of modalities for the treatment for neurological diseases, including chronic pain. Electrical spinal cord stimulation (SCS) refers to an FDA-approved treatment for chronic and neuropathic pain conditions that involves delivering electric current via epidural leads implanted into the spinal cord. This method of treatment is thought to attenuate pain perception via blocking the transmission of nociceptive signaling in the spinal cord dorsal horn according to the Gate Control Theory (1, 2). Conventional SCS, which delivers currents via static electrical pulses with constant stimulation parameters (i.e., ‘tonic’), may introduce paresthesia such as tingling sensations at certain stimulus intensities above perception threshold (3). More recent SCS paradigms that deliver electrical stimuli below perceptual levels (paresthesia-free) have also been shown to achieve analgesic efficacy. Moreover, SCS using time-dynamic pulses (TDPs) is an emerging field of neuromodulation and a promising advancement that, unlike tonic SCS, modulates stimulation pulse parameters with signals that change continuously and automatically (4–6).

While different paradigms of stimulation have shown effectiveness on pain relief, evaluation has often been focused on analgesic effect during the stimulation on period. Our team previously demonstrated that TDPs delivered at paresthesia-free or minimal paresthesia amplitudes significantly reverse allodynia in a rat model of neuropathic pain (4). Specifically, we showed that different TDP stimulations, including sinusoidal rate modulation, stochastic rate modulation, amplitude modulation, and pulse width modulation, significantly reversed allodynia in rats with chronic constriction injury (CCI), when delivered for 60 min. These stimulations were delivered at a baseline amplitude that was below perception threshold, which was expected to induce no or minimal paresthesia. However, in the case of amplitude or pulse width modulation the charge delivered was modulated sinusoidally around the baseline amplitude which could have resulted in minimal paresthesia during the upper cycle of the modulation where the highest intensity takes place. Moreover, SCS reversed an electroencephalography (EEG) signature of spontaneous pain in rats with CCI (4, 7). Our results also indicated that TDPs demonstrated sustained attenuation of hypersensitivity at the end of the 60 min SCS period, suggesting that the analgesic effects of TDPs may extend beyond the hour-long duration tested. Given that SCS is a long-term therapy approach, and there are stimulation on and off periods in clinical settings, it is crucial to evaluate the change or variation in analgesic effect during an extended stimulation on period as well as a stimulation off period. Our current study aimed to extend the timeframe of SCS to investigate the hypothesis that the analgesic effects of TDP persist longer than those of tonic stimulation. Therefore, we sought to compare the efficacy and time course of analgesic responses to TDP stimulation with modulation in amplitude, pulse width, and rate to that of tonic stimulation in the same animal model of CCI. Moreover, we sought to investigate the temporal dynamics of SCS effect (namely, wash-in and wash-out times). Compared to our former study, here we extended the SCS period from 60 min to 90 min for a more comprehensive temporal assessment of the time for producing (i.e., during SCS ‘on’) and maintaining analgesic efficacy (i.e., after SCS ‘off’), including the time to onset and decay of paw withdrawal threshold (PWT) that mirrors respectively the wash-in time to induce analgesia and the wash-out time to lose analgesia. Results from this study support our hypothesis that the analgesic effect from TDP stimulation is prolonged during the stimulation ON period and early after the stimulation is turned off, as compared to tonic stimulation. It should be noted that the baseline pulse parameters were comparable with tonic stimulation, and the modulation parameters were comparable for different TDP. The parameters were chosen without optimization for any setting. Information obtained from this study is clinically valuable and suggest that SCS using time dynamic pulses could potentially produce longer lasting pain relief compared to conventional tonic SCS.

## 2 Methods

### 2.1 Study design

**Figure 1:**
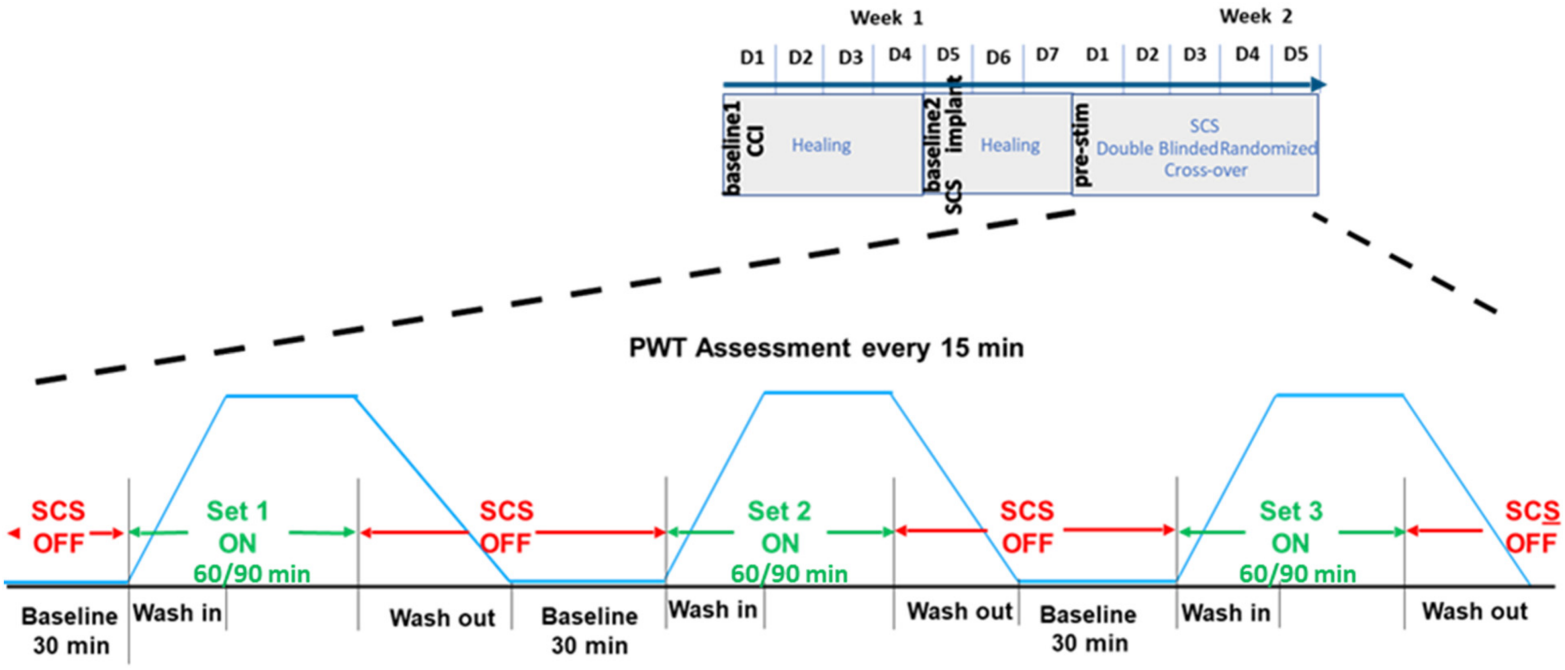
Time course of behavioral paw withdrawal threshold (PWT) measurements in relation to chronic constriction injury (CCI) and spinal cord stimulation (SCS).

**Figure 2:**
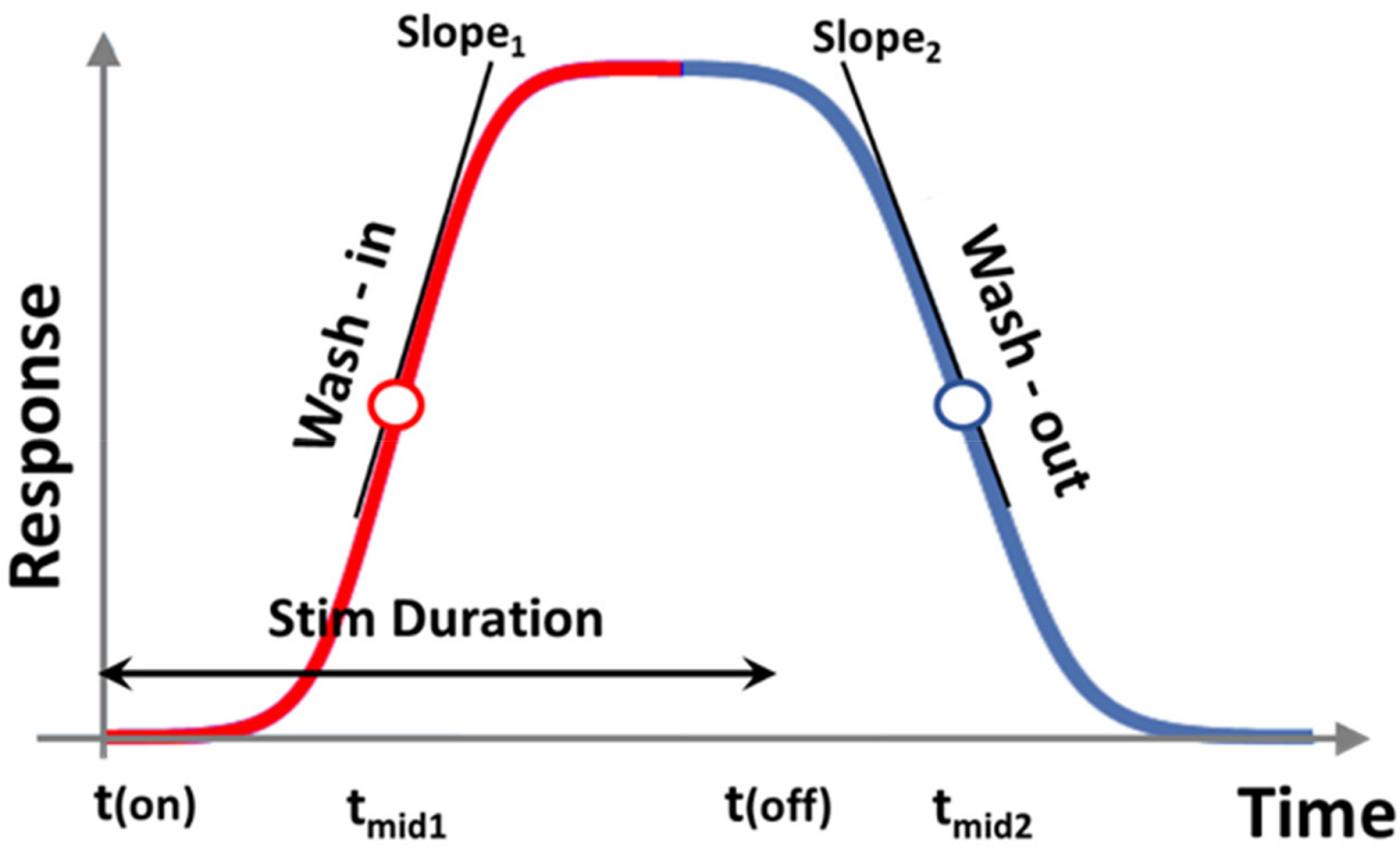
Double sigmoidal curve function for modeling response. *t*_mid1_, *t*_mid2_, *slope*_1_, and *slope*_2_ all indicate different metrics for gauging response onset and decay. *t*_mid1_ and *t*_mid2_ indicate time for response to rise and to decay to half maxima since the start and end of stimulation, respectively. *slope*_1_ and *slope*_2_ determine the speed of growth and speed of decay, respectively.

We conducted a longitudinal, randomized, blinded crossover experiment in *n* = 23 rats, including eight previously reported samples (4), and additional fifteen new samples (Figure 1). For the new samples (*n* = 15), we extended the SCS duration from 60 to 90 min. Behavioral responses to von Frey filaments during SCS and 30 min thereafter were recorded as PWT at 15 min intervals. Each rat received five sessions of SCS including one tonic stimulation session and 4 different TDP stimulation sessions (Amplitude Modulation; Pulse Width Modulation; Sinusoidal Rate Modulation; and Stochastic Rate Modulation) assigned in a random order. The random orders of stimulation sessions for all study animals were pre-generated by computer prior to the study. During the experiments, both the experimenter applying the von Frey test and the data processor analyzing and interpreting the von Frey data were blinded to the von Frey filament strength and order of stimulation sequences. Stimulation parameters for all patterns and measures of perceptual threshold are consistent with those outlined in our previous study (4). The time response curves of PWT per rat and per SCS session were fitted with a double sigmoidal function (Figure 2). We analyzed the time course of PWT responses to estimate the time it takes for the PWT to reach half of the maximum PWT reversal during SCS ‘on’ and the time it takes to decay to half of the maximum PWT reversal after SCS ‘off’. Our results are reported here in accordance with ARRIVE guidelines (https://arriveguidelines.org/).

### 2.2 Experimental animals and surgical operations

Adult (200–300 g) male Sprague Dawley rats were housed under a 12 h light/dark cycle in a temperature and humidity-controlled environment. Food and water were available *ad libitum*. All surgical procedures were performed under deep anesthesia (isoflurane, 2%–2.5%). All the methods were carried out in accordance with the relevant guidelines and regulations. Experiments were approved by Cleveland Clinic Lerner Research Institute Institutional Animal Care and Use Committee (IACUC) and, for the first cohort, by Rhode Island Hospital IACUC. Induction of CCI, implantation of the spinal leads, and configuration and delivery of SCS all followed methods and procedures outlined in our previous study (4).

### 2.3 Responsive curve analysis and temporal evaluation of PWT time course

Paw withdrawal threshold to von Frey filaments were recorded in the awake subjects during SCS ‘on’ (60 or 90 min) and ‘off’ (30 min) at 15 min intervals. The time course of PWT reflected the analgesic effect of SCS, which we assumed follows a biphasic ramp-up after SCS onset and wind-down after SCS ‘off’, modelled as a double sigmoid curve in Figure 2. Hence, we identified the baseline, maximum, and residual PWT responses, and the corresponding timeline when these responses occurred. We adapted a double-sigmoidal model to fit the PWT responses over time using optimization functions provided in MATLAB (MathWorks, MA). Sigmoidal and double sigmoidal curves have been widely used to model dose-response data or biological growth data (8, 9). In our study, the five different types of SCS patterns represent different treatments and the two variable durations of SCS (60 vs. 90 min) represent the dose, while the PWT reversal represents response to treatment.

The target function of the sigmoidal curve fitting was the time course of PWT [i.e., *PWT* (*t*)] change during SCS ‘on’ and after SCS ‘off.’ To account for variations in baseline and maximum PWT reversal, we pre-processed PWT values by subtracting the baseline to obtain the difference in *PWT* (*t*) vs. *PWT* (*t* = 0), which were then normalized to baseline PWT prior to SCS ‘on’ *PWT* (*t* = 0). This normalization procedure removed variations in baseline across individual animals and yielded relative changes from baseline. Most response curves were scaled to a range within (0, 2) post pre-processing. The pre-processed PWT data that served as the unit-less target function *f*_target_ (*t*) is defined as:

$$f_{target}(t)=[PWT(t)-PWT(t_{0})]/PWT(t_{0})$$

The fitting curve was represented as a time dependent function *f*_fit_ (*t*) as:

$$f_{fit}\left(t\right)=\;I\times \frac{1}{1+e^{-a_{1}^{\prime }\left(t-t_{mid1}\right)}}\frac{1}{1+e^{-a_{2}^{\prime }\left(t-t_{mid2}\right)}}$$

where (*I*) was a scaling factor to account for the magnitude of PWT reversal (response); *t*_mid1_ estimates the time at which the response has risen to half of its maximum; *t*_mid2_ estimates the time at which the response has decayed halfway from its maximum to its final value; and the parameters *a*′_1_ and *a*′_2_ determine the steepness of the ramp-up and wind-down phases of the sigmoidal curves, respectively, but do not exactly equal to the slopes of the curve at times *t*_mid1_ and *t*_mid2_. The latter was represented as *slope*_1_ and *slope*_2_, and calculated from the 1st order derivative of the double-sigmoidal function *f*_fit_ (*t*) at times *t* = *t*_mid1_ and *t* = *t*_mid2_. As the target function is unit-less, the slope was calculated in units of 1/min. These metrics represent the ramp-up and wind-down effects, respectively, of analgesia as measured by PWT.

The initial fitting parameter was set at *I* = 1, *a*_1_ = 0.5, *a*_2_ = −0.5, *t*_mid1_ = *t*_stim_on_ + *T*_stim_/4, and *t*_mid2_ = *t*_mid1_ + *T*_stim_, where *t*_stim_on_ = 0, *T*_stim_ = 60 for the first cohort and 90 for the second cohort. The fitting parameters were obtained through minimizing the cost function, which was defined as the summed squared deviation (SSD) of the fit data points to the raw data points via:

$$SSD=\;\overset{N_{t}}{\underset{i=1}{\sum }}\;[\;f_{\mathrm{fit}}(dt(i-1))-f_{\mathrm{target}}(dt(i-1)){]}^{2}$$

where *dt* refers to the time interval between observations (15 min), and *N*_t_ refers to the total number of observations during recording period (*N*_t_ = 7 for the first cohort of 8 samples and *N*_t_ = 9 for the second cohort of 15 samples).

The quality of fitting was evaluated by calculating the percent deviation which is the ratio of the summed square of deviation over the summed square of raw data,

$$Dev\text{\%}=\frac{SSD}{SSR}=\;\frac{\sum_{i=1}^{N_{t}}\;{\left[\;f_{\mathrm{fit}}(dt(i-1))-f_{\mathrm{target}}(dt(i-1))\right]}^{2}}{\sum_{i=1}^{N_{t}}\;{\left[\;f_{\mathrm{target}}(dt(i-1))\right]}^{2}}\times 100\text{\%}$$

where the summed square of the raw data was calculated as:

$$SSR=\;\overset{N_{t}}{\underset{i=1}{\sum }}\;[\;f_{\mathrm{target}}(dt(i-1)){]}^{2}$$

From these fitted models, various temporal metrics were calculated, including *t*_mid1_, *t*_mid2_, and the full-width-half-maximum (FWHM) of the response, which was defined as the percentage of time with response above half of the maximal PWT recovery. Each FWHM was calculated as the duration between *t*_mid1_ and *t*_mid2_, normalized by the total duration of SCS stimulation, i.e., (*t*_mid2_ − *t*_mid1_)/*T*_stim_, where *T*_stim_ = 60 min for the first cohort of 8 samples and *T*_stim_ = 90 min for the second cohort of 15 samples.

### 2.4 Statistical analysis

We performed statistical analysis with native functions in Prism 9 (GraphPad Software, San Diego). We considered *α* = 0.05 as statistically significant (*). Additionally, as we sought to compare TDPs collectively against conventional tonic stimulation as the gold standard (rather than between-pattern comparison, for example amplitude vs. pulse width TDP), we implemented a two-way repeated measures omnibus test (ANOVA for paw withdrawal threshold, mixed-effects model for the double-sigmoidal slope values), followed by multiple comparisons without *post hoc* corrections for behavioral analysis of PWT data. For each pulse pattern, we assessed the time course of PWT under the stimulation using the same pattern across all rats by aggregating the data from both cohorts for a same time period after stimulation onset (post-SCS onset, 0 < *t* ≤ 60) and then performed statistical tests to compare the PWT at each time point post stimulation onset to that obtained at the time point pre-SCS (*t* = 0).

## 3 Results

### 3.1 Sigmoidal curve fitting to time course of PWT

**Figure 3:**
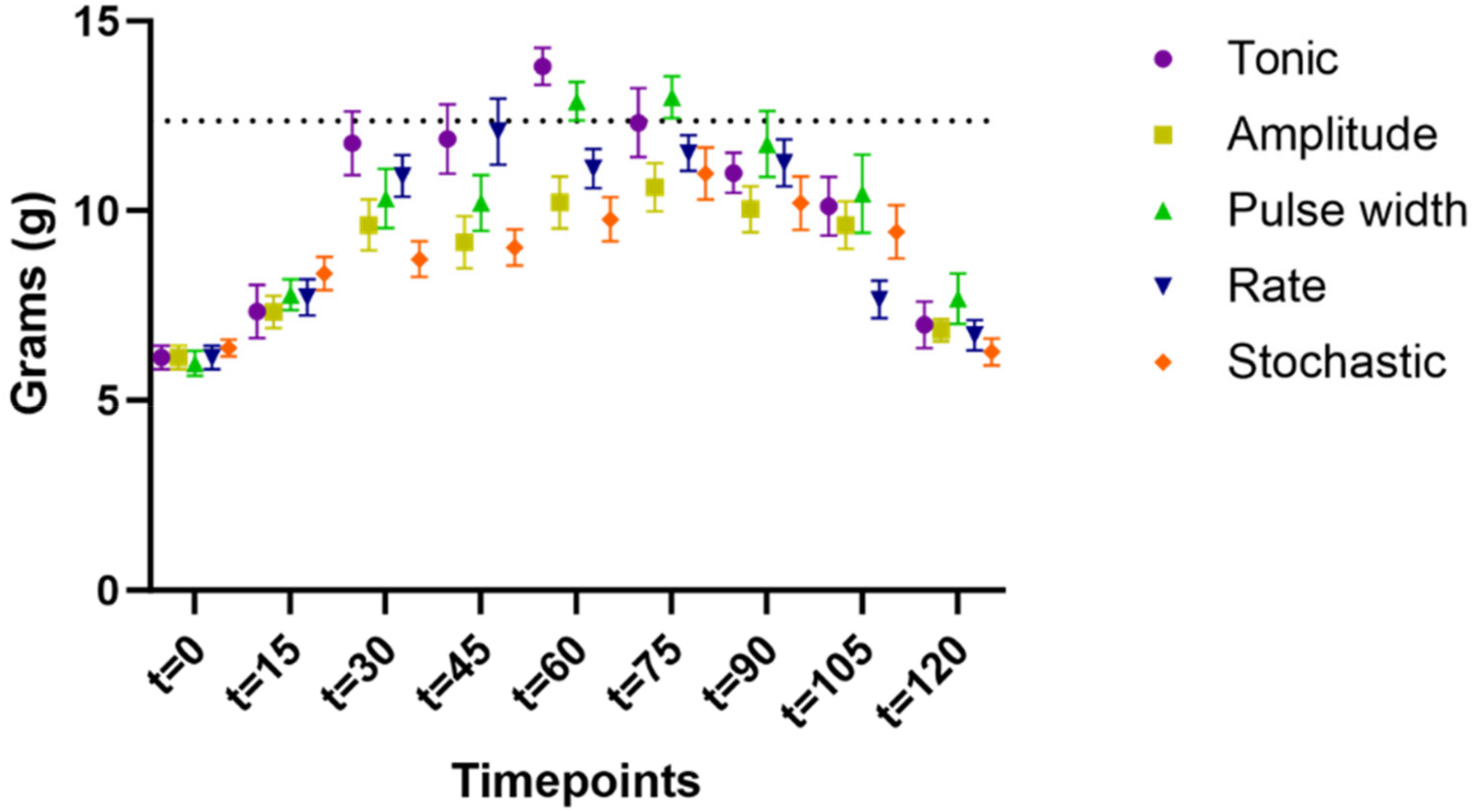
Time course of paw withdrawal threshold (PWT) obtained during SCS using 5 different patterns from cohort #2 (*n* = 15). The PWT data for different stimulation were distinguished by the marker color and shape. Datapoints at each assessment timepoint were plotted slightly offset to reduce overlapping.

Figure 3 shows the time course of PWT obtained during SCS using 5 different patterns from cohort #2 (*n* = 15). PWT time course for cohort #1 (*n* = 8) followed a similar time course as for cohort #2 (4). The time course from both cohorts displayed a typical curve of biphasic response with a ramp-up of response after stimulation was turned on, and wind-down of response after stimulation was switched off, albeit at rates that were different for different stimulation sequences. Sigmoidal curve fitting was performed to each dataset of PWT and analyses were performed on curve fitting parameters and their derivatives. Nine out of 115 fitting datasets were excluded from the analysis based on the following criteria:

(1) When the percent deviation exceeded 50% (*n* = 2); or
(2) When the scaling factor (I) was:
  (a) smaller than 0.1 (indicating that the fitted model demonstrated a change in PWT response of less than 10% from baseline, *n* = 1), or
  (b) negative (indicating that the fitted model ran in the opposite direction of the expected change in PWT response, *n* = 3); or
(3) When both of the aforementioned exclusion criteria were met (*n* = 3).

Of note, no rat had greater than two datasets excluded.

The average percent deviation of fitting for all models (including all samples without exclusions) fell under 15% (tonic: 7.9% ± 2.1%; amplitude: 15.2% ± 4.3%; pulse width: 13.4% ± 4.2%; rate: 13.6% ± 4.2%; stochastic: 12.1% ± 3.4%), and the average percent deviation of fitting after exclusion fell under 10% (except 10.5% for amplitude).

### 3.2 SCS modulation of PWT

**Figure 4:**
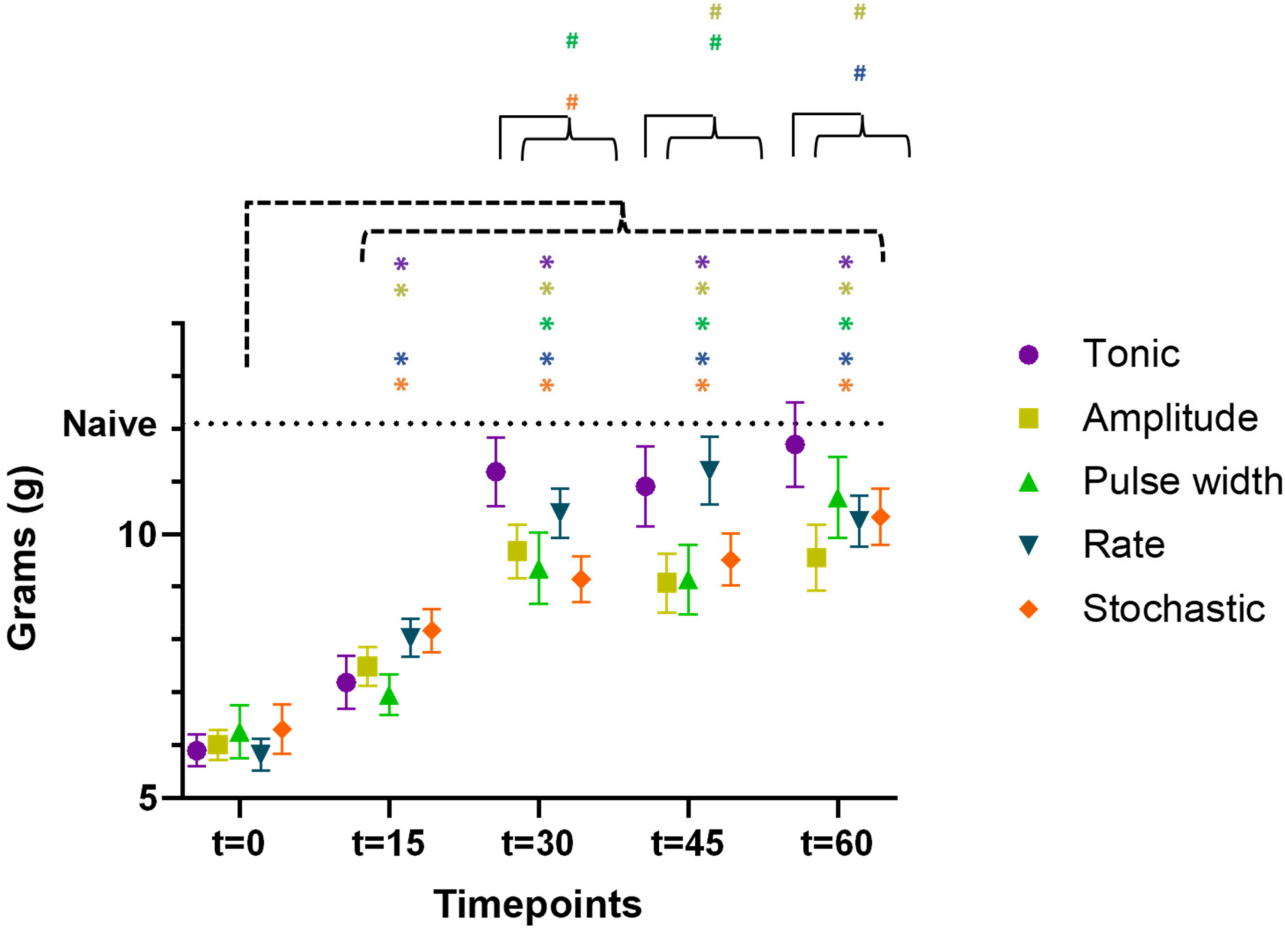
Time course of paw withdrawal threshold (PWT) during the first 60 min of SCS using 5 different patterns (data aggregated from both cohorts, *n* = 23 rats), demonstrating the wash-in of stimulation effect. The PWT data for different stimulation were distinguished by the marker color and shape. Datapoints at each assessment timepoint were plotted slightly offset to reduce overlapping. Asterisk * marked the statistical significance in PWT as compared to baseline (*t* = 0 min) using two-way ANOVA for repeated measures. Hashtag # marked the statistical significance in comparison of PWT across 5 different SCS patterns at the same time point of *t* = 30, 45, and 60 min, respectively (both cohorts, *n* = 23 rats).

Aggregating data from both cohorts (*n* = 23) for the time course of PWT during first 60 min of SCS were shown in Figure 4, demonstrating wash-in of stimulation effect. All five types of SCS stimulation reduced allodynia towards pre-CCI values, such that PWT were elevated during the first 60 min of stimulation as compared to pre-stimulation (*t* = 0), according to the timeline shown in Table 1. Asterisk * in Figure 4 marked the statistical significance in PWT as compared to baseline (*t* = 0 min) using two-way ANOVA for repeated measures. All types of stimulation significantly reversed PWT at all time points *t* = 15, 30, 45, and 60 min, except for pulse width modulation at *t* = 15 min, where the increase in PWT did not reach significant levels. For each timepoint between *t* = 0 and 60 min at 15 min intervals, we compared PWT during the four TDP stimulation vs. tonic stimulation, whereby the PWT was not significantly different at *t* = 0 and *t* = 15 min. However, difference was observed at time point of *t* = 30, 45 and 60 min (marked with hashtag # in Figure 4). PWT was significantly higher during tonic stimulation as compared to some TDP stimulations, in particular at *t* = 30 min (11.2 ± 0.6 g) after pulse width (9.4 ± 0.7 g, *p* < 0.05) and stochastic modulation (9.1 ± 0.4 g, *p* < 0.01), at *t* = 45 min (10.9 ± 0.8 g) after amplitude (9.1 ± 0.6 g, *p* < 0.05) and pulse width modulation (9.1 ± 0.7 g, *p* < 0.05), and at *t* = 60 min (11.7 ± 0.8 g) after amplitude (9.6 ± 0.6 g, *p* < 0.05) and rate modulation (10.2 ± 0.5 g, *p* < 0.05).

**Table 1:** PWT were significantly elevated during stimulation at *t* = 15, 30, 45 and 60 min as compared to pre-stimulation PWT (*t*_o_ = 0), except for pulse width modulation at *t* = 15 min.

Stimulation type and *PWT* (*t*_0_ = 0) (g)	Time *t* (min)	*PWT* (*t*) (g)	*p*-value
Tonic 5.9 ± 0.3	15	7.2 ± 0.5	*p* < 0.05
	30	11.2 ± 0.6	*p* < 0.001
	45	10.9 ± 0.8	*p* < 0.001
	60	11.7 ± 0.8	*p* < 0.001
Amplitude 6.0 ± 0.3	15	7.5 ± 0.4	*p* < 0.01
	30	9.7 ± 0.5	*p* < 0.001
	45	9.1 ± 0.6	*p* < 0.001
	60	9.6 ± 0.6	*p* < 0.001
Pulse width 6.3 ± 0.5	15	7.0 ± 0.4	*p* = 0.22
	30	9.4 ± 0.7	*p* < 0.01
	45	9.1 ± 0.7	*p* < 0.001
	60	10.7 ± 0.8	*p* < 0.001
Rate 5.8 ± 0.3	15	8.0 ± 0.4	*p* < 0.001
	30	10.4 ± 0.5	*p* < 0.001
	45	11.2 ± 0.6	*p* < 0.001
	60	10.2 ± 0.5	*p* < 0.001
Stochastic 6.3 ± 0.5	15	8.2 ± 0.4	*p* < 0.01
	30	9.1 ± 0.4	*p* < 0.001
	45	9.5 ± 0.5	*p* < 0.001
	60	10.3 ± 0.5	*p* < 0.001

### 3.3 Impact of extending SCS duration from 60 to 90 min on PWT

**Figure 5:**
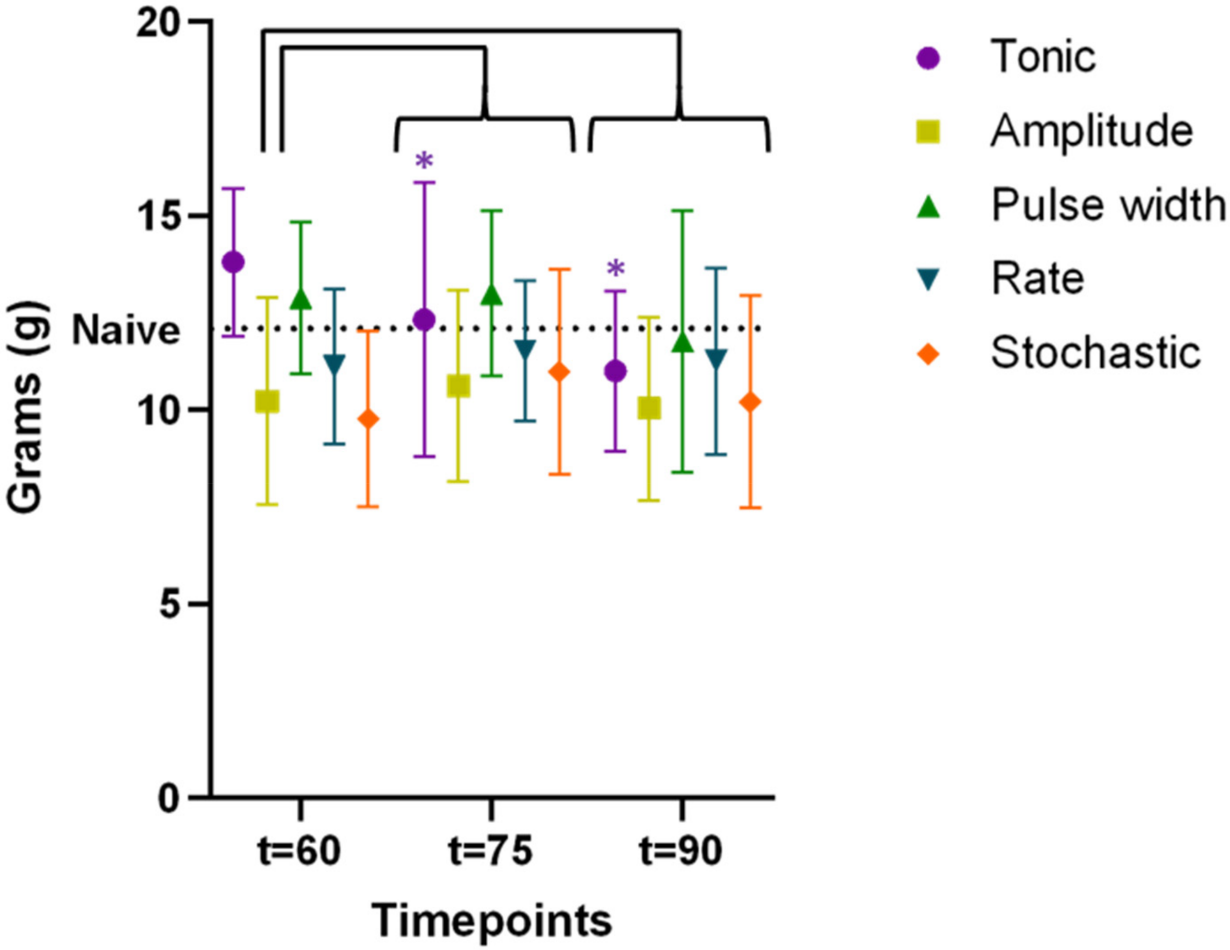
Time course of paw withdrawal threshold (PWT) during extended stimulation period up to *t* = 90 min (data available for the cohort #2 only, *n* = 15 rats), demonstrating the sustaining of stimulation effect. The PWT data for different stimulation were distinguished by the marker color and shape. Datapoints at each assessment timepoint were plotted slightly offset to reduce overlapping. The difference in PWT as compared to that measured at *t* = 60 min were assessed using two-way ANOVA test. Only tonic SCS exhibited significantly degraded efficacy at *t* = 75 min, with further decline at *t* = 90 min, whereas PWT during TDPs remained stable during the same time period.

For the second cohort (*n* = 15) with extended stimulation period, time course of PWT during extended period t = 60–90 min were shown in Figure 5, demonstrating the sustaining of stimulation effect. All TDP stimulations maintained comparable anti-allodynic effects at *t* = 60, 75 and 90 min. However, these effects significantly degraded for tonic stimulation at *t* = 75 min (12.3 ± 0.9 g, *p* < 0.05) and further at *t* = 90 min (11.0 ± 0.5 g, *p* < 0.01) compared to *t* = 60 min (13.8 ± 0.5 g), as displayed in Figure 5. This analysis of extended analgesic effects was restricted to the stimulation interval from 60 to 90 min.

### 3.4 Wash-in and wash-out of analgesic effects as measured by PWT

**Figure 6:**
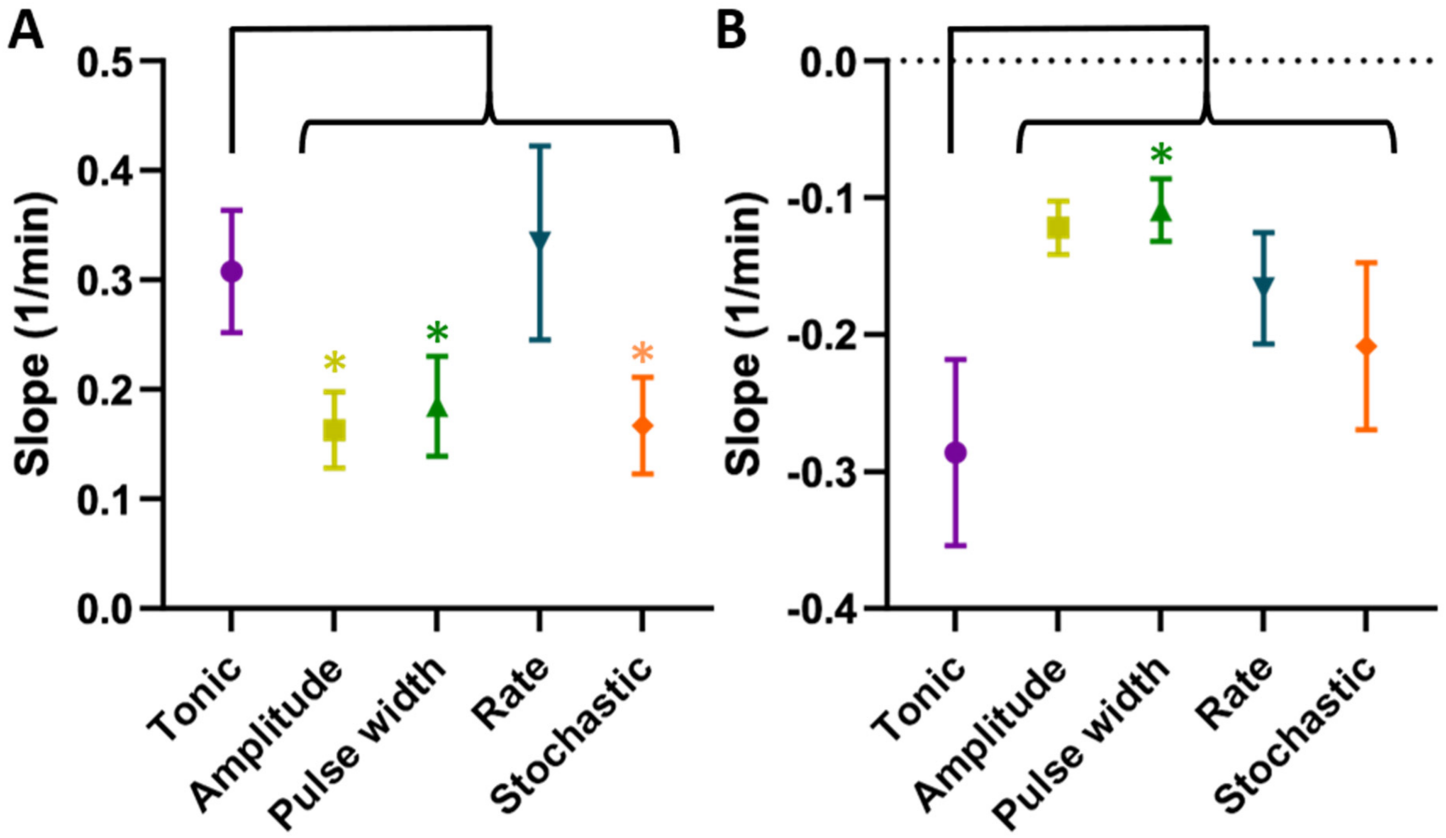
**(A)** *Slope*_1_ (speed of growth or “wash-in”). TDPs (amplitude, pulse width, and stochastic rate) had significantly slower wash-in times compared to tonic stimulation, with rate modulation achieving comparable speed. **(B)** *slope*_2_ (speed of decay or “wash-out”). TDP stimulations had slower wash-out times compared to tonic SCS, with pulse width reaching significance and having the longest wash-out. Statistical significance of the difference was tested using multiple comparisons without *post hoc* corrections for behavioral analysis.

To evaluate the time course of ramp-up and wind-down of the anti-allodynic effects, we compared slope values for each TDP stimulation to tonic stimulation, whereby a greater (more positive) *slope*_1_ indicated a faster wash-in and a greater (less negative) *slope*_2_ indicated a slower wash-out. The *slope*_1_ values (Figure 6A) were significantly lower for amplitude modulation (0.16 ± 0.03 min^−1^, *p* < 0.05), pulse width modulation (0.18 ± 0.05 min^−1^, *p* < 0.05) and stochastic modulation (0.17 ± 0.04 min^−1^, *p* < 0.05) compared to tonic stimulation (0.31 ± 0.06 min^−1^). However, *slope*_1_ for rate modulation (0.33 ± 0.09 min^−1^) was not different from that of tonic stimulation. The *slope*_2_ values (Figure 6B) were higher (i.e., slower wash-out) for amplitude modulation (−0.12 ± 0.02 min^−1^), rate modulation (−0.17 ± 0.04 min^−1^) and stochastic modulation (−0.21 ± 0.06 min^−1^) compared to tonic stimulation (−0.29 ± 0.07 min^−1^), with pulse width modulation reaching significance (−0.11 ± 0.02 min^−1^, *p* < 0.05).

Mean Full-Width-Half-Maximum (FWHM) of the response curve for each TDP stimulation was higher compared to tonic stimulation, although the difference did not reach significance (Table 2), noting that FWHM values for TDP stimulations were closer in range relative to tonic stimulation.

**Table 2:** Full-Width-Half-Maximum (FWHM) by pattern set across both cohorts (*n* = 23), which reflects the percentage of the total time analgesic response is demonstrated for a given pattern.

Pattern set	Full width half maximum (FWHM) (Mean ± SEM)
Tonic	0.779 ± 0.067
Amplitude	0.904 ± 0.057
Pulse width	0.892 ± 0.055
Rate	0.925 ± 0.037
Stochastic	0.889 ± 0.053

FWHM for pattern set of Amplitude, Pulse Width, Rate, and Stochastic were closer relative to each other and were all higher than to that for Tonic (Mean ± SEM).

## 4 Discussion

In this study, we investigated the anti-allodynic effects of five different SCS stimulations consisting of four dynamically modulated pulse patterns and one conventional tonic stimulation, at paraesthesia-free or minimal paraesthesia amplitude level, in a rat model of neuropathic pain. We used paw withdrawal threshold (PWT) to Von Frey stimulus as a surrogate measure of analgesic efficacy. Such methods have been widely applied before to assess clinical analgesia and the effects of SCS in animal models (10–12). One advantage of this study is the blinded cross-over design with a randomized testing order, enabling a more robust comparison of different pulse patterns of stimulation.

This study in particular evaluated the time course of PWT during a SCS period up to 90 min for a more comprehensive temporal assessment for producing (i.e., during SCS ‘on’) and maintaining analgesic efficacy (i.e., after SCS ‘off’), including the wash-in time and the wash-out time. In spinal cord stimulation, wash-in time refers to the period it takes for the stimulation to start producing clinically significant pain relief after the stimulation is turned on. Evaluating wash-in time helps determine how quickly the patient is responding to the therapy, thus guiding when to adjust the stimulation parameters for optimal results. Conversely, wash-out time refers to the duration it takes for the pain relief effect to diminish after the stimulation is turned off. Evaluating wash-out time is crucial for understanding the lasting effect of the therapy during the post-stimulation period and ensuring that patients receive sustained pain relief even during periods when stimulation is off.

Consistent with our previous study, our results demonstrate that TDPs and tonic stimulation significantly reversed allodynia within thirty minutes of SCS onset and throughout the first 60 min of stimulation after SCS onset. New findings from this study with the extended stimulation period showed that the sustainability of the effect could differ as stimulation was extended. Overall, analgesic effects from all four TDP stimulations were maintained over the extended stimulation period from 60 to 90 min, while analgesic efficacy from tonic stimulation significantly degraded gradually over the same extended period, even though tonic stimulation achieved higher analgesic effects during the period between 30 and 60 min. These findings may reflect tolerance to tonic stimulation or desensitization of the neural circuits in the spinal cord to ongoing, constant pulse stimulation (13, 14), which is one of the limiting complications of SCS therapy and is characterized by a progressive decrease of pain relief over time when a repeated treatment is presented. This change in response causes the loss of therapeutic efficacy over time (15). Sometimes it is described as “Failed SCS Syndrome” (FSCSS) (16), which in some cases leads to explant of the device. In a retrospective chart review of therapy related explants after SCS by Van Buyten et al. (17), over 50% of explants were performed due to the loss of analgesic benefit from SCS over time. While in some cases the loss of efficacy can be attributed to the loss of coverage, in other cases patients experience decreased efficacy despite no loss of coverage. The underlying mechanism of stimulation tolerance is not yet fully understood, but could be attributed to a variety of reasons, such as physiological changes (15), or neural adaptation to constant stimulation (18, 19). Evaluating changes in analgesic effect during the stimulation helps assess the built-up of tolerance to the therapy. The sustained effect from dynamic stimulation as compared to that from static stimulation suggests that technologies incorporating dynamic variations in stimulation parameters may have potential to reduce or delay the build-up of tolerance or neural adaptation, consequently leading to enhanced therapeutic longevity.

Further investigation into the temporal dynamics of analgesic efficacy (using a novel model fitting method to approximate PWT based on a sigmoidal curve function) revealed delayed, but prolonged analgesic effects for TDPs relative to tonic stimulation. Traditionally, PWT is analysed by assessing the averaged PWT response curve over time, evaluating the turning points and line trends for changes in effect. While the response to the stimulation is a continuous function over time, a typical Von Frey test can only measure PWT at discrete time points. This limitation makes the assessment of turning points highly sensitive to noise. Fitting the discrete PWT values measured with a mathematical model is a novel approach. To our knowledge, this is a first attempt to use model fitting for PWT analysis. Sigmoidal functions have been widely used to describe the dose-response relationship or growth patterns in many physiological or pharmacological studies, as they enable the evaluation of an induced effect in a biological system as a function of the level of the stimulus (8). The PWT time course recorded before, during, and after SCS application, follows a biphasic process ramping up after stimulation onset and winding down after stimulation ends, making double sigmoidal function a good candidate model. The benefits of approximating the discrete assessments of PWT with fitted mathematical models were four-fold: (1) it smoothed the variations in behavioural responses due to the limited temporal resolution and the noise inherent to animal behaviour; (2) it allowed quantitative estimates for depicting the time course of said responses; (3) it enabled comparisons between two sets of data from distinct cohorts; and (4) it provided a mathematical tool to identify outliers.

The overall percent deviation (<10% after exclusion of a small set of outliers, i.e., 9 out of 115) suggested that the selected model mathematically approximated the individual time courses of PWT within reasonable deviation. The difference in average slope values obtained from the fitted models for different stimulations matched the trend observed in the time course of averaged raw PWT data, suggesting the model reasonably captured the temporal characteristics of the PWT time course. Moreover, FWHM values for all TDP stimulations exceeded those of tonic stimulation and compared favourably with each other, which is consistent to the observation in the plots of PWT time course suggesting that analgesic effects produced by TDP stimulation may be sustained for longer. However, we do acknowledge that there could still be limitations in our model fitting. Given that the PWT data is a discrete measure at relatively sparse time points, and for the Von Frey test there is only a limited set of possible outcome values, the number or the density of the data points can restrict the applicable models and affect the quality of the model fitting. Our model might not represent a mathematically perfect fit and other phasic models may be employed as candidate models in future studies.

The mechanisms of action for tonic and virtually all forms of SCS are not fully understood, however, the recruitment of mechanical Aβ-fiber afferents most likely “gates” the transmission of nociceptive signals (2, 20, 21). Given that only tonic stimulation elicited tolerance over prolonged stimulation periods in our study, it is possible that TDP-modulated SCS more closely replicates the irregular and asynchronous activity in physiological states of the dorsal horn (22–24). For example, amplitude and pulse width modulated stimulation potentially mimic the physiological encoding of mechanosensory stimuli (25, 26). Meanwhile, rate-modulated stimulation may replicate the firing rate of slowly adaptive primary afferents, thereby altering the reflex response (22). It may also induce long-term potentiation mediated by NMDA receptors or glial cells (27–29). Investigation of these and other mechanisms and how they may differ between and within TDPs is warranted, enabling further optimization of the different TDP stimulations in clinical settings.

## Data Availability

The raw data supporting the conclusions of this article will be made available by the authors, without undue reservation.
